# A novel magnetic resonance imaging–based classification of subscapularis muscle atrophy: reproducibility and clinical relevance

**DOI:** 10.1016/j.xrrt.2026.100720

**Published:** 2026-03-19

**Authors:** Wataru Sahara, Takehito Hirose, Masataka Kiyomoto, Kosuke Kuratani, Yuki Kotani, Seiji Okada

**Affiliations:** a Department of Orthopaedic Surgery, The University of Osaka Graduate School of Medicine, Osaka, Japan; b Department of Rehabilitation Medicine, The University of Osaka Hospital, Osaka, Japan; c Department of Orthopaedic Sports Medicine, Yukioka Hospital, Osaka, Japan; d Department of Orthopaedic Surgery, Japan Community Healthcare Organization Osaka Hospital, Osaka, Japan; e Department of Orthopaedic Surgery, Toyonaka Municipal Hospital, Osaka, Japan

**Keywords:** Subscapularis, Muscle atrophy, New classification, Reproducibility, Clinical relevance, Muscle strength

## Abstract

**Background:**

Muscle atrophy and fatty degeneration of rotator cuff are critical factors influencing surgical indications for rotator-cuff tears. However, no widely accepted method exists for the evaluation of subscapularis (SSC) atrophy. Based on our previous clinical experience, we hypothesized that SSC atrophy extends from the cranial side and proposed a novel magnetic resonance imaging–based classification of SSC atrophy. This study aimed to investigate the validity of the proposed classification and its relationship with clinical parameters.

**Methods:**

In total, 70 patients (72 shoulders; mean age, 62.3 ± 11.5 years) who underwent arthroscopic rotator-cuff repair or reverse shoulder arthroplasty were included. SSC atrophy was evaluated preoperatively on T1-weighted oblique sagittal images in the Y-shaped view, in which the lateral scapular spine and coracoid base were visualized. Atrophy was classified as follows: none (superior margin of the SSC extends beyond the anteroinferior edge of the coracoid base), mild (superior margin tapers below this edge), moderate (anteroposterior width is reduced at the scapular body–spine intersection), or severe (anteroposterior width is reduced at the inferior tip of the scapular body). Reproducibility was assessed by 2 shoulder surgeons using intraclass correlation coefficients (ICCs). To evaluate clinical relevance, correlations between this classification and the SSC-tear size, fatty degeneration, and internal rotation strength were examined. In addition, sensitivity and specificity to detect the presence of a comma sign (retracted SSC tendon stump) were calculated using this classification.

**Results:**

The classification was applicable to all cases. Intrarater and inter-rater reliability was excellent (ICC [1, 2] = 0.89, ICC [2, 1] = 0.85, respectively). Higher atrophy grades of SSC was significantly correlated with greater tear size, higher fatty degeneration grade, and lower internal rotation strength (Spearman ρ = 0.65, 0.64 and −0.42, respectively, *P* < .001, all). When grade ≥1 (mild atrophy) was defined as abnormal, the presence of the comma sign was predicted with 59% sensitivity and 82% specificity.

**Discussion:**

This classification, which was based on the craniocaudal extension of SSC atrophy, utilizes clear bony landmarks that enable a high reproducible and simple evaluation. Its significant associations with tear size, fatty degeneration, and internal rotation strength support its value as a clinically relevant indicator. Moreover, its ability to predict the presence of the comma sign suggests that the classification reflects both muscle atrophy and tendon retraction.

**Conclusion:**

The proposed magnetic resonance imaging–based classification of SSC atrophy demonstrated high reproducibility and validity, effectively reflecting tear size, tendon retraction, and internal rotation strength and thereby establishing its clinical relevance.

## Graphical abstract

**Figure:**
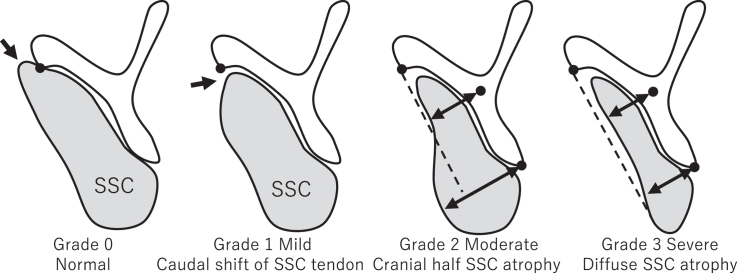

Rotator-cuff tears are a major cause of shoulder dysfunction; among these tears, subscapularis (SSC) tendon tears account for approximately 27–62% of all cases.^1^^,^^8^^,^^19^ Particularly, when a SSC tear is added to a supraspinatus (SSP)–infraspinatus (ISP) tear, the superior migration of the humeral head may occur, potentially resulting in impaired shoulder elevation.^4^^,^^22^^,^^26^ In such cases, arthroscopic rotator-cuff repair (ARCR) may be insufficient, and reverse shoulder arthroplasty (RSA) is often required as an alternative.^28^

Muscle atrophy and fatty degeneration of the rotator cuff are critical indicators for determining the feasibility of primary repair^6^^,^^12^^,^^13^ and are strong predictors of long-term functional recovery.^5^^,^^20^ Therefore, the quantitative evaluation of rotator-cuff muscle quality on imaging has significant clinical importance. Fatty degeneration is widely assessed using the Goutallier classification, which can be applied to all rotator-cuff muscles, including the SSC, SSP, ISP, and teres minor.^9^ In contrast, the assessment of muscle atrophy has largely been limited to the SSP, with the tangent sign proposed by Zanetti et al^31^ and the occupation ratio described by Thomazeau et al^27^ being well-established methods. With respect to SSC atrophy, Warner et al^30^ described a classification where the entire SSC muscle gradually atrophies. However, subsequent studies have reported that, in cases of SSC tears, atrophy occurs predominantly in the cranial half.^3^^,^^24^ This disparity may explain why Warner's classification has not gained widespread clinical adoption.

Therefore, we propose a novel magnetic resonance imaging (MRI)-based classification of SSC muscle atrophy using oblique sagittal images, based on the hypothesis that SSC atrophy preferentially involves the cranial portion and extends toward the caudal portion. This study aimed to validate the clinical applicability and reproducibility of this new classification system. Specifically, we hypothesized that SSC atrophy would be correlated with clinically relevant parameters, including SSC tendon tear size, fatty degeneration, and internal rotation strength.

## Materials and methods

### Study participants

This retrospective study included 73 patients (75 shoulders), who underwent ARCR or RSA for rotator-cuff tears at our institution between 2020 and 2022. Of these, 3 patients (3 shoulders), in whom SSC muscle atrophy could not be adequately evaluated on MRI, were excluded, leaving 70 patients (72 shoulders) in the final study cohort. The mean age at surgery was 62.3 ± 11.5 years (range, 24–81 years). The cohort comprised 43 men and 29 women, with a mean height of 162.0 ± 9.4 cm (range, 140–182 cm) and a mean weight of 67.1 ± 14.0 kg (range: 40–106 kg) (Table I).

**Table I:** Demographic data.

Item	Number/Value
Number of patients and shoulders	70/72
Age (yr)	62.3 ± 11.5
Male/female	43/28
Condition of rotator cuff	
Partial tear	
Only SSP	7
SSC-SSP	5
Complete tear	
Only SSP	14
Only SSC	1
SSC-SSP	22
SSP-ISP	4
SSC-SSP-ISP	19
Distribution of SSC tear (Lafosse classification)	
Normal	25
I:Partial lesion of superior one-third	26
II:Complete lesion of superior one-third	8
III:Complete lesion of superior two-thirds	9
IV:Complete lesion of tendon but head centered and ≤ Goutallier stage 3	4
V:Complete lesion of tendon but eccentric head and ≥ Goutallier stage 3	0
Type of surgery (ARCR/RSA)	67/5

*SSC*, subscapularis; *SSP*, supraspinatus; *ISP*, infraspinatus; *ARCR*, arthroscopic rotator cuff repair; *RSA*, reverse shoulder arthroplasty.

The condition of the rotator cuff was diagnosed based on preoperative magnetic resonance imaging (MRI) and intraoperative findings. The tear size of subscapularis (SSC) tendon was graded according to the classification proposed by Lafosse (Grades I–V).^14^

This study was approved by the Institutional Review Board of the Academic Clinical Research Center of Osaka University (approval ID: 22548). Informed consent for the use of clinical data was obtained from all patients prior to surgery.

### Assessment of MRI

**Figure 1:**
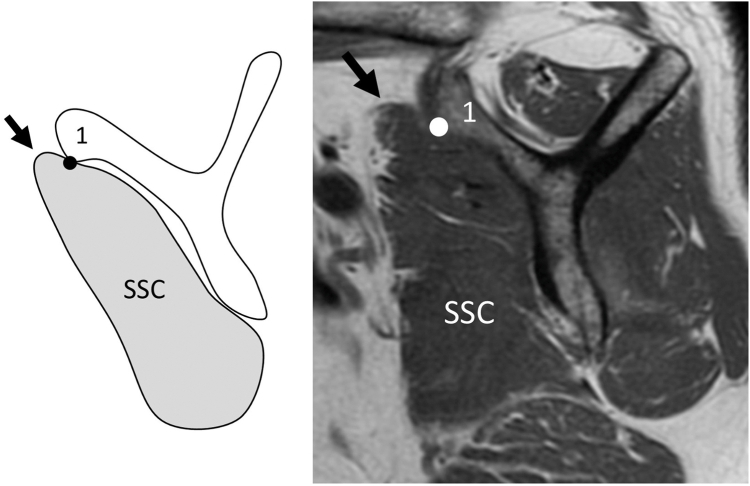
Subscapularis (SSC) atrophy, Grade 0 (Normal). The Y-shaped view was selected on T1-weighted oblique sagittal magnetic resonance imaging (MRI), defined as the slice visualizing both the base of the coracoid process and the lateral border of the scapular spine. In Grade 0, the superior margin of the SSC contour is located cranial to the anteroinferior edge of the coracoid base (*Point 1*).

All patients underwent MRI within three months prior to surgery using a 1.5-Tesla scanner (SIGNA Explorer, GE Healthcare, Chicago, IL, USA). The upper limb was fixed in neutral shoulder rotation. Three imaging planes were acquired: the axial plane (horizontal to the trunk), the oblique coronal plane (parallel to the scapular body on the axial view), and the oblique sagittal plane (perpendicular to the scapular body on the axial view). T1-weighted oblique sagittal images (TR/TE = 500/10; slice thickness = 4 mm; field of view = 160 × 270 mm) at the level where the lateral edge of the scapular spine and the base of the coracoid process were visualized (Y-shaped view) were used to evaluate SSC muscle atrophy and fatty degeneration (Fig. 1).

**Figure 2:**
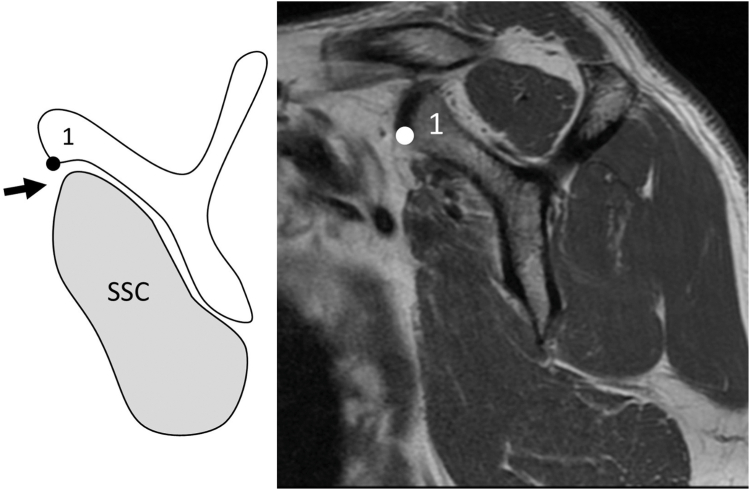
Subscapularis (SSC) atrophy, Grade 1 (mild: caudal shift of SSC tendon). In Grade 1, the superior margin of the SSC contour is located caudal to *Point 1*, indicating a slight caudal shift of the SSC tendon relative to the coracoid base. However, this grade is not accompanied by a reduction in the anteroposterior width of the SSC unlike that in higher grades (Fig. 3). *Point 1*: the anteroinferior edge of the coracoid base.

**Figure 3:**
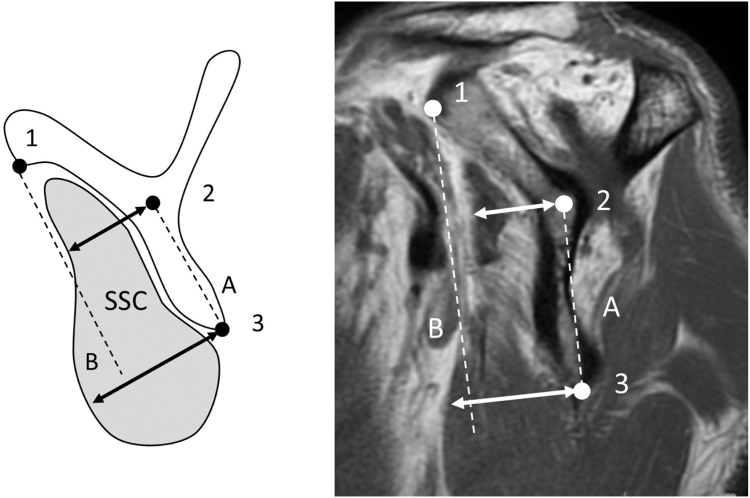
Subscapularis (SSC) atrophy, Grade 2 (moderate: cranial half SSC atrophy). In Grade 2, the anterior margin of the SSC muscle belly is located posterior to *Line B* at *Point 2*, demonstrating a localized reduction in the anteroposterior width of the SSC at this level, while the more caudal portion remains preserved. *Point 2*: intersection of the coracoid base, scapular spine, and scapular body. *Point 3*: inferior tip of the scapular body. *Line A*: line along the scapular body. *Line B*: line parallel to Line A passing through *Point 1*.

**Figure 4:**
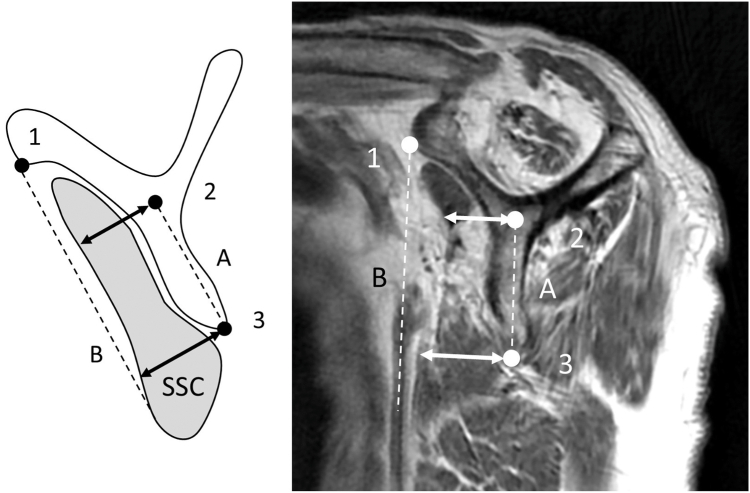
Subscapularis (SSC) atrophy, Grade 3 (severe: diffuse SSC atrophy). In Grade 3, the anterior margin of the SSC muscle belly is located posterior to *Line B* at *Points 2* and *3*, demonstrating a diffuse reduction in the anteroposterior width of the SSC muscle belly.

SSC muscle atrophy was classified as follows: cases in which the superior margin of the SSC was located cranially to the anteroinferior edge of the coracoid base were classified as grade 0 (normal, Fig. 1); those in which the superior margin was located caudally to this level were classified as grade 1 (mild; caudal shift of SSC tendon, Fig. 2); cases in which the anteroposterior width of the SSC was reduced at the intersection of the coracoid process, scapular body, and scapular spine were classified as grade 2 (moderate; cranial half SSC atrophy, Fig. 3); and cases in which the anteroposterior width was reduced at the level of the inferior tip of the scapular body were classified as grade 3 (severe; diffuse SSC atrophy, Fig. 4).

Two independent shoulder surgeons, blinded to all patient background information, performed the assessments. One observer repeated the evaluation after a 3-month interval to assess intrarater reliability. Furthermore, the fatty degeneration of the SSC was evaluated in the Y-shaped view using the modified Goutallier classification proposed by Fuchs et al,^7^ graded from 0 to 4.

### Surgical findings

**Figure 5:**
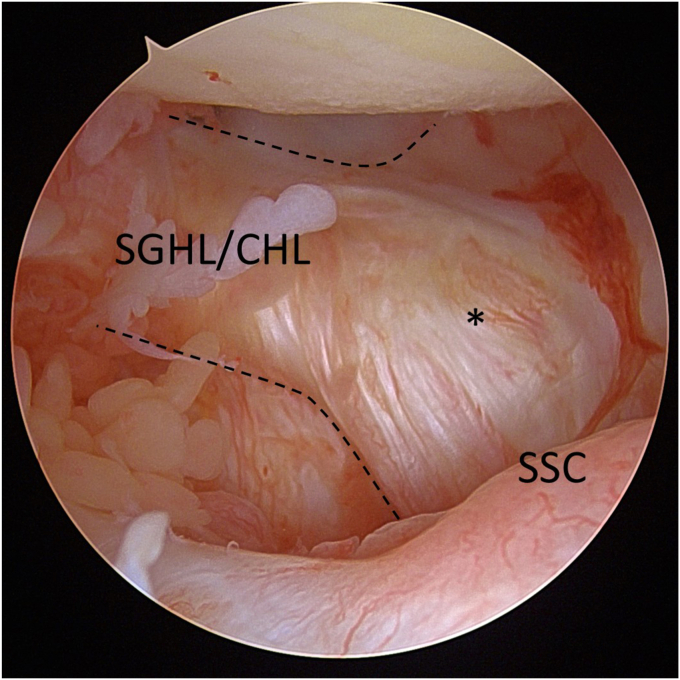
Arthroscopic observation of the comma sign. Arthroscopic view through the posterior portal of the right shoulder in the lateral decubitus position. The comma sign (*asterisk*) described by Lo et al^16^ represents an arc-shaped structure formed by the superior glenohumeral ligament (*SGHL*) and coracohumeral ligament (*CHL*) complex. This structure indicates the superolateral corner of the torn subscapularis (*SSC*) tendon.

ARCR was performed with all patients in the lateral-decubitus position. SSC lesions were assessed through the standard posterior portal. Because the quantitative evaluation of tendon retraction of the SSC is difficult, the presence or absence of the comma sign—a marker indicating the superolateral corner of the retracted SSC tendon stump—was recorded (Fig. 5).^16^ In addition, the size of the rotator-cuff tear was carefully examined. When necessary, the bicipital groove was incised to confirm whether the superficial layer of the SSC remained attached to the lesser tuberosity. The SSC-tear size was graded according to Lafosse classification (grades I–V), and normal tendons were classified as grade 0.^14^

RSA was performed in the beach-chair position using the deltopectoral approach. The superficial layer of the SSC remaining on the lesser tuberosity were carefully released along the anterior margin of the bicipital groove; the tendon was meticulously inspected to determine whether it remained attached to the lesser tuberosity. Based on these intraoperative findings, the SSC-tear size was graded according to Lafosse classification system.^14^ The presence or absence of the comma sign could not be confirmed in 5 cases during RSA.

### Clinical findings

Clinical evaluations included assessments of pain, range of motion, and muscle strength. Among these parameters, muscle strength was considered the most direct indicator of SSC function; therefore, internal rotation strength was selected as the representative clinical measure.

To measure the internal rotation strength, we adopted the Napoleon test-based technique, which showed both a strong correlation with SSC-tear size and high reproducibility in our previous study.^21^ Measurements were performed by 2 physical therapists, each with over three years of experience in shoulder rehabilitation. Patients were seated with their backs against the wall, palms placed on the abdomen and elbows positioned at the anterior–posterior midpoint of the trunk. One examiner stabilized the patients' shoulder and hand position, while the other applied resistance using a hand-held dynamometer (microFET 2; Nihon Medix Co., Ltd., Chiba, Japan) placed on the lateral side of the elbow. The patients were instructed to push their elbow forward, and maximal isometric strength was recorded. Measurements were performed 3 times, and the mean value was used for analysis. To adjust for differences in body size, the measured values were converted to kilograms and normalized by body weight.

### Statistical analysis

To assess the reproducibility of the new SSC-atrophy classification, intraclass correlation coefficients (ICCs) were calculated. Intrarater reliability was evaluated using ICC (1, 2), and inter-rater reliability using ICC (2, 1). ICC values were interpreted as follows: 0.81–1.0, excellent; 0.61–0.80, high; 0.41–0.60, moderate; 0.21–0.40, fair; and ≤0.20, poor.

To evaluate the clinical relevance of the SSC-atrophy classification, we investigated its correlations with age, sex, tear size (Lafosse classification^14^), fatty degeneration (Fuchs classification^7^), presence of comma sign, and internal rotation strength. Continuous variables, including age, Lafosse classification, Fuchs classification, and internal rotation strength, were analyzed using the Kruskal–Wallis test and Spearman rank correlation coefficient. Categorical variables, including sex and the presence of the comma sign, were analyzed using the chi-square test. Furthermore, SSC atrophy of grade ≥1 was defined as abnormal, and its relationship with the presence of the comma sign was evaluated using the chi-square test. Sensitivity and specificity for the presence of the comma sign were calculated.

ICCs were calculated using IBM SPSS Statistics (version 21; IBM Corp., Armonk, NY, USA), whereas chi-squared tests and multiple regression analyses were performed using JMP Pro (version 17; SAS Institute Inc., Cary, NC, USA). The significance level was set at *P* < .05.

A power analysis was conducted to determine the required sample size using R (version 4.5.1; R Foundation for Statistical Computing, Vienna, Austria). For reliability assessment (ICC), the sample size was calculated by setting the expected ICC at 0.90, null-hypothesis ICC at 0.70, power at 0.90, and significance level (α) at 0.05, resulting in a required sample size of 30 shoulders. For Spearman rank correlation coefficient analysis, the required sample size was calculated with an effect size of 0.5 (medium), a significance level (α) of 0.05, and power of 0.95, resulting in a required sample size of 45 shoulders.

## Results

### Demographic data and tear characteristics

This study included 70 patients (72 shoulders). The SSC condition was classified as follows: 25 shoulders were normal, 26 had partial tears, 8 had complete tears involving less than one-third of the lesser tuberosity attachment, 9 had complete tears involving up to two-thirds, and 4 had complete tears involving more than two- (Table I).

### Applicability and reproducibility of SSC-atrophy classification

The proposed classification was applicable to all cases. SSC atrophy was classified as grade 0 in 47, grade 1 in 11, grade 2 in 9, and grade 3 in 5 shoulders (Table II). Intrarater reliability showed an ICC (1, 2) value of 0.89, and inter-rater reliability an ICC (2, 1) value of 0.85, indicating excellent reliability. Discrepancies between raters were observed in 15 of the 72 shoulders (21%). A one-grade discrepancy occurred in 14 cases as follows: in 5 shoulders, one rater assessed grade 0 while the other assessed grade 1; in 7 shoulders, the discrepancy was between grades 1 and 2; and in 2 shoulders, between grades 2 and 3. A two-grade discrepancy was observed in only 1 case, in which one rater judged grade 1 and the other judged grade 3.

**Table II:** Group comparisons according to subscapularis (SSC) atrophy classification.

SSC atrophy	0		1	2	3	*P* value
Number (shoulders)	47		11	9	5	
Age (yr)	62 ± 11.7		59 ± 13.1	64.1 ± 8.6	69 ± 9.9	.42
Sex (male/female)	M 32/F 15		M 6/F 5	M 5/F 4	M 0/F 5	.03
Tear size (Lafosse)	0.66 ± 0.81		1.1 ± 0.54	2.7 ± 0.87	3.6 ± 0.55	<.0001∗
Fatty degeneration (Fuchs)	0.06 ± 0.25		0.27 ± 0.64	1.0 ± 0.87	2.2 ± 0.44	<.0001†
Comma sign (presence/none)	P	9	5	6	2	.0018
	N	37	6	2	0	
Internal rotation strength	7.5 ± 0.51%		5.5 ± 1.1%	4.1 ± 1.1%	3.6 ± 1.5%	.0079‡

Internal rotation strength was measured using a hand-held dynamometer according to the Napoleon test. To adjust for differences in body size, strength values were normalized to body weight.

∗ Significant differences were observed for all pairwise comparisons except between grades 0 and 1 and between grades 2 and 3.

† Significant differences were observed for all pairwise comparisons except between grades 0 and 1.

‡ A significant difference was observed only between grades 0 and 2; the comparison between grades 0 and 3 showed a trend toward significance (*P* = .08).

### Correlations between SSC-atrophy classification and clinically relevant parameters

Age did not differ significantly across SSC-atrophy classification groups, whereas sex distribution differed significantly among groups (*P* = .03; Table II). The proportion of female patients increased with increasing SSC-atrophy grade.

The SSC-atrophy classification showed a moderate positive correlation with the SSC-tear size assessed by the Lafosse classification^14^ (Spearman ρ = 0.65, *P* < .0001), indicating that the SSC-atrophy grade worsened as the SSC-tear size increased. Group comparisons also demonstrated significant differences (*P* < .0001; Tables II and III).

**Table III:** Relationship between subscapularis (SSC) atrophy and the SSC-tear size.

SSC atrophy Tear size	0	1	2	3	Total (72)
Normal	24	1	0	0	25
I	17	8	1	0	26
II	4	2	2	0	8
III	2	0	5	2	9
IV	0	0	1	3	4

Similarly, the SSC-atrophy classification was moderately positively correlated with fatty degeneration according to the Fuchs classification^7^ (ρ = 0.64, *P* < .0001), with more severe fatty degeneration being associated with more severe SSC atrophy. Significant differences were also observed in group comparisons (*P* < .0001; Tables II and IV).

**Table IV:** Relationship between subscapularis (SSC) atrophy and the SSC fatty degeneration.

SSC atrophy FD	0	1	2	3	Total (72)
0	44	9	3	0	56
1	3	1	3	0	7
2	0	1	3	4	8
3	0	0	0	1	1
4	0	0	0	0	0

*FD*, grade of fatty degeneration.

In contrast, the SSC-atrophy classification showed a moderate negative correlation with internal rotation strength (ρ = − 0.42, *P* = .0003), indicating that more severe SSC atrophy was associated with decreased internal rotation strength. Group comparisons likewise revealed significant differences (*P* = .0079; Table II).

### Diagnostic performance of SSC-atrophy classification for the comma sign

The chi-square test showed that the distribution of the comma sign differed significantly according to the SSC muscle-atrophy grade (*P* = .0018; Table II). When SSC atrophy of grade ≥1 was defined as abnormal, 13 of the 22 shoulders with a comma sign showed abnormal SSC atrophy, yielding a sensitivity of 59%. In contrast, among the 45 shoulders without a comma sign, 37 showed normal SSC atrophy, corresponding to a specificity of 82% (Table V).

**Table V:** Relationship between subscapularis (SSC) atrophy and presence of the comma sign.

SSC atrophy Comma sign	Abnormal	Normal	Total (67)
Presence	13	9	22
None	8	37	45

SSC atrophy of grade ≥1 was defined as abnormal.

## Discussion

In this study, we focused on the craniocaudal extension of SSC muscle atrophy and developed a novel MRI-based classification system reflecting this pattern. We validated its reproducibility and clinical relevance. This classification demonstrated excellent intrarater and inter-rater reliability and moderate correlations with tear size, fatty degeneration, and internal rotation strength. Furthermore, when SSC atrophy of grade ≥1 was defined as abnormal, the sensitivity and specificity for the presence of the comma sign were 59% and 82%, respectively. These findings indicate that the use of this classification in the preoperative evaluation may serve as an adjunctive indicator of a retracted SSC tendon and help predict the need for SSC repair preoperatively.

Regarding the evaluation of SSC atrophy, Warner et al (2001)^30^ proposed a four-grade SSC-atrophy classification using the Y-shaped MRI view, defining grades based on the position of the SSC muscle belly relative to a line drawn from the coracoid edge to the inferior tip of the scapular body. With increasing atrophy severity, the SSC cross-sectional area diminishes, and its contour, which initially extends beyond this triangular region, becomes gradually confined within it. However, this pattern of atrophy is rarely observed in clinical practice. In contrast, recent quantitative studies have shown that SSC atrophy extends predominantly from the cranial side. Scheibel et al^23^ reported shortening of the craniocaudal length and cranial anteroposterior width of the SSC muscle belly after open stabilization surgery in patients with recurrent shoulder dislocation. These morphological changes correlated with higher positivity rates on the belly-press and belly-off tests. Similarly, Seppel et al^24^ compared isolated SSC tears with normal shoulders and found that the ratio of the cranial half to the total SSC cross-sectional area was significantly smaller in the tear group. Bartl et al^3^ further reported that a smaller cranial anteroposterior width was associated with a higher rate of positive belly-press tests after SSC repair. Based on these observations, we designed a new classification system that evaluates shortening of the craniocaudal length and reduction in the cranial anteroposterior width.

Seppel et al^24^ and Bartl et al^3^ have reported methods for evaluating SSC morphology by calculating the ratio of the anteroposterior width or cross-sectional area of the cranial portion to that of the caudal portion of the SSC. However, these methods are time-consuming and are primarily effective in cases wherein the caudal portion of the SSC is preserved; these methods may underestimate atrophy in cases, wherein the entire SSC muscle is atrophied. To develop a simple classification that is not influenced by the degree or pattern of SSC morphology, we adopted the coracoid process as a bony landmark on the Y-shaped view. Because this method relies on clear anatomical landmarks, it has high reproducibility and can be easily applied in routine clinical practice without requiring advanced expertise. Our study demonstrated excellent intrarater reliability (ICC = 0.89) and inter-rater reliability (ICC = 0.85), values that are acceptable for clinical application. Moreover, in 14 of 15 discrepant cases, disagreement was limited to a single grade. All discrepancies occurred in cases, wherein the SSC contour overlapped the reference lines used to differentiate between the grades. Between grades 0 and 1, discrepancies were observed when the most cranial portion of the SSC overlapped the anteroinferior margin of the coracoid base. Between grades 1 and 2, and between grades 2 and 3, discrepancies occurred when the anterior border of the SSC overlapped a line drawn parallel to the scapular body through the anteroinferior margin of the coracoid process. These borderline cases highlight the inherent difficulty of categorical classification when anatomical structures are located close to predefined reference lines. These results indicate that this classification is a practical and objective tool for evaluating SSC atrophy.

This classification can be interpreted as an assessment system that reflects both retraction of the SSC tendon stump and muscle atrophy. SSC atrophy of grade ≥1 was associated with the presence of the comma sign, which represents medial retraction of the SSC tendon stump. Jo and Shin^11^ reported that the cross-sectional areas of the SSP and ISP increased on the Y-shaped view immediately after cuff repair, indicating that medially retracted muscles were repositioned laterally postrepair. The cranial two-thirds of the SSC consists predominantly of tendon tissue, whereas the caudal one-third is composed mainly of muscle.^2^ Given that SSC tears typically extend cranially, the loss of tension in the cranial tendon may lead to caudal displacement and early morphological changes corresponding to grade 1 in our classification. When SSC atrophy of grade ≥1 was defined as abnormal, the sensitivity and specificity for the presence of the comma sign were 59% and 82%, respectively. According to a systematic review by Malavolta et al,^17^ MRI diagnosis of SSC tendon tears demonstrated 68% sensitivity and 90% specificity, with lower sensitivity for partial tears than for complete tears. Several authors have noted that the evaluation of partial tears of the superior SSC tendon on conventional axial views is challenging; modifications in arm positioning or imaging planes have been proposed to improve detection.^18^^,^^25^^,^^29^ In the present study, abnormal findings of grade ≥1 showed low sensitivity but specificity exceeding 80% for the comma sign, suggesting that that the presence of abnormal findings should prompt consideration of the repair of the retracted SSC tendon during surgical planning.

Because no significant differences were observed in tear size or fatty degeneration between grades 0 and 1 of SSC atrophy (Table II), grade 1 should be interpreted not as muscle atrophy but as a finding that warrants attention to the presence of an SSC tear accompanied by a comma sign. In contrast, grades 2 and 3 capture a reduction in the anteroposterior width of the SSC and are considered to reflect muscle atrophy. Once SSC atrophy reaches grade ≥2, the tear size is grade ≥2 (corresponding to a complete tear involving more than one-third of the SSC), and fatty degeneration is grade ≥1, indicating a condition that reflects muscle atrophy accompanied by degenerative changes. Furthermore, this classification showed a negative correlation with internal rotation strength. Although clinical evaluation generally includes pain, range of motion, muscle strength, and imaging findings, the present cohort included cases with concomitant SSP and ISP tears; therefore, pain and range of motion were considered to be of limited value as SSC-specific functional assessments. Accordingly, the evaluation of internal rotation strength was considered a useful indicator that more directly reflects SSC function. Given the difficulty in capturing SSC-specific clinical symptoms, this classification may serve as a clinically important indicator reflecting the pathophysiology of the SSC.

One strength of this classification is its high versatility, as it does not require specialized MRI techniques or scanning conditions and can be applied in virtually any facility. It also offers practical clinical usefulness because precise measurements of tendon length or muscle area are unnecessary. Furthermore, it is visually intuitive and demonstrates high reproducibility. A second strength is that it allows simultaneous assessment of the following two important changes: SSC muscle atrophy and tendon retraction. This information may be valuable for preoperative evaluation and for predicting postoperative muscle function, and it may help prevent missed diagnoses of retracted SSC tears. Additionally, this classification is expected to be useful for postoperative evaluation and longitudinal follow-up.

**Figure 6:**
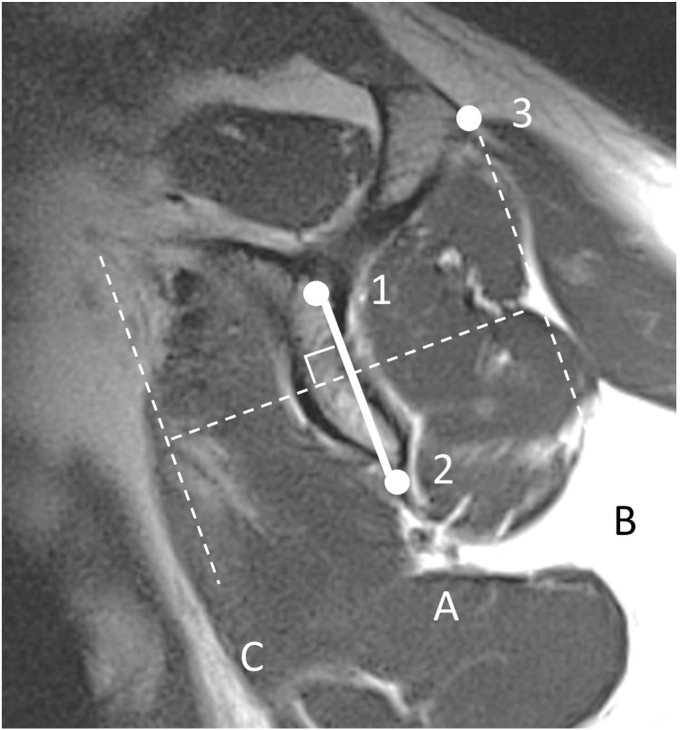
Alternative method for evaluating subscapularis (SSC) atrophy. When neither the base of the coracoid process nor the lateral border of the scapular spine can be visualized within a single slice, and a typical Y-shaped view is therefore unobtainable, an alternative evaluation method is proposed for measuring the anteroposterior width of the SSC muscle belly. *Point 1*: intersection of the coracoid process, scapular spine, and scapular body. *Point 2*: inferior tip of the scapular body. *Point 3*: posterior edge of the scapular spine. *Line A*: line drawn along the scapular body. *Line B*: line parallel to Line A passing through Point 3. *Line C*: line parallel to Line A positioned midway between Lines A and B. Using Line C as a reference, it would be possible to determine whether the anteroposterior width of the SSC is reduced.

This study has several limitations. First, most cases involved combined rotator-cuff tears, including the SSP and ISP, and only a few involved isolated SSC tears. Therefore, this study may not be sufficient to establish a clear causal relationship between SSC muscle atrophy and clinically relevant parameters. Second, because this study was based on preoperative evaluations, the tear size, muscle atrophy, and fatty degeneration are interrelated factors that may influence one another. Although the present study demonstrated a correlation between muscle atrophy and internal rotation strength, internal rotation strength may be affected by multiple factors, including tear size and fatty degeneration. To more accurately evaluate the impact of SSC atrophy, future studies should examine the relationship between atrophy and internal rotation strength in postoperative cases without retears. Third, this study did not include normal shoulders as a control group, and therefore it was not verified whether all normal cases would be classified as grade 0 using this classification. Morphological variations of the coracoid process have been reported, particularly with regard to the shape of the coracoid tip.^10^^,^^15^^,^^32^ Although the coracoid base was selected as the bony landmark in the present study, some variability has also been reported in the angle between the coracoid base and the long axis of the glenoid, as well as in the length of the coracoid base.^32^ These morphological variations may potentially influence the applicability of this classification. Finally, this was a single-center study, and the MRI acquisition parameters may vary across institutions. Differences in the angles of oblique sagittal imaging or MRI sequences could affect the reproducibility of the classification. In particular, the angle of the oblique sagittal plane is critical: if both the base of the coracoid process and the lateral border of the scapular spine are not captured within a single slice, a typical Y-shaped view cannot be obtained, and the apparent anteroposterior width of the SSC muscle belly may be altered. When the base of the coracoid process was not clearly visualized, the coracoid base could not be used as a reference. In such cases, a reference line was established anteriorly, parallel to the scapular body, at a distance equal to that between the scapular body and the posterior border of the scapular spine, and the anteroposterior width of the SSC was compared relative to this line (Fig. 6). Alternatively, if the caudal portion of the SSC shows no apparent atrophy, the width at the inferior tip of the scapular body could be used as a reference. Future studies should include multicenter validation and investigate the relationship between this classification and postoperative outcomes.

## Conclusions

The novel MRI-based classification of SSC atrophy proposed in this study demonstrated excellent reproducibility. Its significant correlations with tear extent, tendon retraction, and internal rotation strength support its validity and clinical relevance as a reproducible tool for preoperative assessment.

## Disclaimers:

Funding: Wataru Sahara received funding from the 10.13039/501100001691Japan Society for the Promotion of Science (JSPS, Japan) KAKENHI Grant Number JP25K12478. The immediate family has not received any financial payments or other benefits from any commercial entity related to the subject of this article.

Conflicts of interest: The authors, their immediate families, and any research foundations with which they are affiliated have not received any financial payments or other benefits from any commercial entity related to the subject of this article.
